# Carers’ experiences of assistive technology use in dementia care: a cross sectional survey

**DOI:** 10.1186/s12877-021-02417-1

**Published:** 2021-08-25

**Authors:** Vimal Sriram, Crispin Jenkinson, Michele Peters

**Affiliations:** Health Services Research Unit, Nuffield Department of Population Health, University of Oxford, Richard Doll Building, Old Road Campus, Oxford, OX3 7LF UK

**Keywords:** Assistive technology, Dementia, Informal carers, Quality of life

## Abstract

**Background:**

Assistive Technology (AT) supports persons with dementia and their carers (family, friends and neighbours), yet little is known about experiences and the impact of AT on carers. We report on an exploratory survey that examined the types, uses, costs and impact of AT on carers as well as their quality of life.

**Methods:**

A cross-sectional survey using the Carers Assistive Technology Experience Questionnaire collected data from carers in the UK, who used at least one AT in the previous year and provided more than 10 h of care for a person with dementia, living at home. Carers completed the questionnaire online or on paper and information on AT, socio-demographic details, and Short-Form Health Survey (SF-12) data were collected. Descriptive and inferential statistics were used to report results and draw conclusions.

**Results:**

Data from 201 carers was analysed. Smartphones and tablet computers were the most frequently used AT. AT were used predominantly for safety, communication, and reminders. Carers usually make decisions on buying and continued use of AT. Multiple AT devices were used in the care of persons with dementia and number of AT used was associated with perceived satisfaction. Satisfaction with AT was not related to age, living arrangements and relationship of carers. From the SF-12, Mean Physical Component Score was 49.19 (95%CI- 47.75 to 50.63) and Mental Component Score was 45.37 (95%CI- 43.93 to 46.80). Women, carers in the 46–65 age group and carers who were not extremely satisfied with AT had lower MCS scores. Carers who lived with the person with dementia and older carers had lower PCS scores.

**Conclusions:**

Carers report that AT has a beneficial impact. Carers use multiple ATs, perceive AT to be satisfactory and recommend AT use to others. To support carers, we recommend establishment of centrally funded information sources and a loan store for AT. Further research on incremental addition of AT and changes to formal/paid care because of using AT should be undertaken. Practitioners, academics, manufactures and policy makers should consider the experiences of carers in research, development and use of AT to facilitate improved community living of people with dementia.

**Supplementary Information:**

The online version contains supplementary material available at 10.1186/s12877-021-02417-1.

## Background

Dementia is a progressive disorder and a public health priority [1, 2]. Some estimates suggest a worldwide societal cost of dementia at approximately US$1 trillion, with this cost doubling by 2030 [3]. A vast amount of the care for persons with dementia who live at home is provided by informal carers (family, friends and/or neighbours) [4, 5]. Within the UK approximately 700,000 informal carers (hereafter referred to as carers) support persons with dementia [5]. With an aging population, this demand for time and support from carers is likely to grow [6]. Assistive technology (AT) could support people with dementia and their carers within the community [7–9]. AT can be defined as: “any item, piece of equipment, product or system that is used to increase, maintain or improve the functional capabilities and independence of people with cognitive, physical or communication difficulties” [10]. During the COVID-19 pandemic [11] and associated restrictions [12, 13], technology has been suggested as a source for social and professional support, communication and safety for persons with dementia and carers [14]. In spite of technology being viewed as a pervasive solution to supporting carers and persons with dementia to live for longer in the community [15–17] there have been very few attempts to understand the experiences of carers, who use and support the use of AT; and their perceptions of the impact of these ATs [9, 18]. Carers of persons with dementia are in the unique position of using their views regarding AT to suggest or even decide on the access and use of AT [19, 20], yet very little is known about the experiences of carers in using AT and what, if any, impact AT has on carers’ wellbeing. Additionally, there continues to be a lack of understanding of the number of AT available and their uses; the ethical implications of choice and continued use of certain AT for privacy and confidentiality; and the perceived satisfaction with and benefits of use of AT from the perspective of carers [21]. Information for this study was gathered using the Carers Assistive Technology Experience Questionnaire (CATEQ) [22]. The CATEQ was created to provide a broader understanding of the experiences of carers using or supporting a person with dementia to use AT. This survey aims to understand the current use, satisfaction and impact of using AT among carers of persons with dementia.

### Study objectives

#### This study investigated

1. the types and use of AT by carers in the support of persons with dementia;
2. the outlay costs, monthly on-going costs and perceived value for money of AT;
3. the perceived impact of AT on carers;
4. the general physical and mental health of carers of persons with dementia who use AT.

##### Ethics and patient and public involvement

This study was approved by the University of Oxford Central University Research Ethics Committee (Reference number: R57703/RE002). No personal identifiable information of participants is reported in this paper. This study is part of a larger research project which has a patient and public advisory group that meets twice a year. This group consists of two carers of persons with dementia and a person with dementia (all living in England). This group reviewed the final version of CATEQ submitted for ethical approval. This group has also committed to support dissemination of study results to other patient involvement groups and their wider networks.

## Methods

### Study design

This study is part of a larger sequential explanatory mixed-method study [23] exploring carers’ experience and impact of AT use in dementia care. This manuscript describes the quantitative phase of the mixed-method study. We conducted a cross-sectional survey between April to July 2020, using the CATEQ. Data was collected by giving potential participants the option of completing the questionnaire electronically through an anonymous link using the electronic survey platform Qualtrics [24] or by post with the printed questionnaire mailed to participants who requested it, with a pre-paid return envelope. A consent question was included at the start of the questionnaire and informed consent was required for online respondents to continue with the survey. Postal versions included the consent question as part of the paper questionnaire and, in addition, participants were instructed to not send back the questionnaire if they did not consent to participating in the survey. We have used STROBE cross sectional reporting guidelines to structure this manuscript [25].

### Participants

Participants were recruited using online databases via the Join Dementia Research website [26], which had more than 48,000 registered volunteers from across the UK at the time recruitment for this study and Oxford Dementia and Aging Research (OxDARE) [27], which had more than 6000 registered volunteers at the time of recruitment for this study. To increase participation from carers from different ethnic backgrounds, requests were made through health care professionals who prescribe AT for persons with dementia. Participants were carers of persons with dementia based in the United Kingdom. The inclusion criteria were: adult carers - family, friends or neighbours - providing at least 10 h of care (e.g. shopping, leisure, personal care, finance) per week to a person with dementia who lives in their own home, with the carer living together with or away from the person with dementia; carers needed to have used at least one AT device at home in the previous year and be able to complete the questionnaire in English.

### Questionnaire

#### Carers Assistive Technology Experience Questionnaire (CATEQ)

The development of the questionnaire is described elsewhere [22], briefly it involved developing items for the questionnaire based on a systematic review [9, 28] and qualitative study [21]; designing and conducting cognitive interviews to test items of the questionnaire; iterative revision of questionnaire based on the cognitive interviews; and final testing on volunteers of the patient and public advisory group. The questionnaire contains questions on types and uses of AT, support provided by the AT, impact of using AT and questions to collect socio-demographic information; with free-text spaces for respondents to qualify their answers if necessary. The time needed to complete the questionnaire was less than 30 min. The full questionnaire is available (supplementary file 1).

#### SF-12

The survey also included the SF-12 Health Survey (version1) [29–31]. The SF-12 contains items covering physical functioning, social functioning, role functioning (physical and mental), vitality, bodily pain, mental health and general health. The SF-12 generates two summary scores: The Physical Component Score and the Mental Component Score (PCS and MCS respectively). The PCS and MCS are generated using norm-based methods and are standardised, using scores from the general population [29, 32], to have a mean of 50 (SD 10). A higher score indicates better quality of life.

#### Independent variables

Carer socio-demographic variables were collected to describe participants. These included age, sex, employment status, marital status, ethnicity, education level, annual family income, living arrangements and relationship with the person with dementia.

#### Dependent variables

For objective (1) - The types and use of AT by carers in the support of persons with dementia - the types and uses of the AT were explored using a list of AT as well as open ended questions asking participants to record AT not listed. For objective (2) - Identify outlay costs, monthly on-going costs and perceived value for money of AT - participants were asked to provide approximate expenditure in Great British Pounds for the initial purchase and monthly ongoing costs of using the AT, as well as indicating who paid for the AT. For objective (3) - Perceived impact of AT for carers of persons with dementia - carers indicated perceived impact of AT in reducing harm, additional time for carers, reducing stress and managing anxiety, concerns about privacy and confidentiality, how AT meets their needs and overall satisfaction with AT. For objective (4) General physical and mental health of cares of persons with dementia who use AT - quality of life of carers who participated was explored with data from the SF-12 in addition to questions on impact of caring on coping and their health.

### Statistical methods

Data analyses were carried out using IBM SPSS Statistics version 26. Descriptive statistics in the form of frequency distributions, percentages, means, standard deviations, and medians were used to examine the types of AT used, what the AT was used for, costs of the AT, perceived value for money and satisfaction with the AT. Bivariate analyses involving a chi-square test, a Mann-Whitney U test and a Kruskal-Wallis test were conducted to examine differences in socio-demographic variables such as education level, living arrangements and relationship status between respondents and SF-12 MCS and PCS scores. An independent-Samples Kruskal-Wallis Test was used to analyse perceived value for money and total costs and monthly costs of use of AT. The level of significance was set at *p* < 0.05 for all analyses.

## Results

A total of 215 questionnaires were returned by respondents. Sixty-six completed questionnaires were returned from a total of 85 participants who requested for the postal questionnaire, with participants based in England, Scotland, Wales and Northern Ireland. A further 149 questionnaires were returned using the online anonymous link on the Qualtrics survey platform. Of these, 3 respondents declined to give consent at the start of the online questionnaire and did not proceed further and were excluded. A further 11 respondents had not completed the questionnaire further than listing the AT used and so their data were excluded. This left a total of 201 participants after exclusions.

### Description of participants

There were 131 (65.2%) women and 65 men (32.3%) with 1 participant self-identifying as non-binary and 4 missing values. Participants’ age ranged from 33 to 92 with a mean age of 62 (SD 12) with most participants between 46 and 65 years (*n* = 105; 52.2%) followed by participants between 66 and 85 years (*n* = 74; 36.8%). Participants were predominantly white (*n* = 186; 92.5%), currently married (*n* = 158; 78.6%) and with a university degree (*n* = 127; 63.2%). Participants were children of a person with dementia (*n* = 110; 54.7%) or a spouse of a person with dementia (*n* = 72; 35.8%) with others caring for in-laws, uncles, and stepparents. For those willing to disclose annual family income (*n* = 153; 76.1%), most participants earned between £10,000 - £ 40,000 (*n* = 86; 42.7%). A fuller description of the participants is available in Table 1.

**Table 1:** Participant characteristics

		N	%
Sex	Women	131	65.2
	Men	65	32.3
	Other	1	0.5
Living arrangements	Living with person with dementia	103	51.2
	Living away from person with dementia	98	48.8
Ethnicity	White	186	92.5
	Indian/Indian British	4	2
	Mixed/multiple ethnic groups	3	1.5
	Other	1	0.5
Marital status	Single	17	8.5
	Married/civil partnership	158	78.6
	Divorced/legally dissolved civil partnership	22	10.9
	Widowed/surviving partner	3	1.5
Highest level of education	Secondary school	8	4.0
	College (further education)	58	28.9
	Undergraduate university degree	76	37.8
	Postgraduate university degree	51	25.4
	Other	8	4.0
Annual family income	Less than £10,000	7	3.5
	£10,001 - £40,000	86	42.7
	£40,001 - £70,000	49	24.4
	Greater than £70,000	11	5.5
	I do not wish to say	47	23.4
Relationship with person with dementia	Parent	110	54.7
	Sibling	3	1.5
	Friend	2	1.0
	Neighbour	1	0.5
	Spouse	72	35.8
	Grandparent	3	1.5
	Other	10	5.0
Age (Minimum – Maximum); Mean (SD)	33–92 Years; 61.67 (12.07)		

### Types and uses of AT

Participants reported using a wide variety of AT (Table 2). Frequently used AT were smart phones (*n* = 91; 45.5%) and tablet computers (*n* = 90; 45.0%) followed by video communication systems (*n* = 89; 44.5%) and dementia clocks (*n* = 82; 41.0%). Less frequently used AT included stair lifts, electric toothbrushes, and hoists. Carers used between 1 to 13 different ATs, with 37.8% carers using less than 4 ATs and 62.2% carers using 5 or more ATs and 6 carers using just 1 AT device. There was no difference in numbers of AT used or abandoned to the age of the carer. AT were predominantly used for safety (*n* = 157; 78.5%) and communication (*n* = 132; 66.0%), followed by AT used to support memory and provide reminders (*n* = 125; 62.5%). AT were least used for managing everyday spending (*n* = 8; 4.0%) and for activities of daily living such as supporting eating, washing etc., (*n* = 17; 8.5%). Among those participants (*n* = 88) who stated that some AT devices that were no longer in use, pendant alarms (*n* = 35; 41.2%) and audio books (*n* = 22; 25.9%) were the most frequently abandoned AT devices. The main reason why AT were abandoned were reported to be because the person with dementia was no longer able to use them (*n* = 69; 81.2%).

**Table 2:** Types of assistive technology

Type of assistive technology in use	N (% of participants)	AT no longer in use N (% of participants)
Assistive robot(s)	1 (0.5%)	
Flood detector	2 (1.0%)	
Robotic pet(s)	2 (1.0%)	
Satnav in car	2 (1.0%)	
Bath lift	2 (1.0%)	
Electric toothbrush	3 (1.5%)	
Stair lift	4 (2.0%)	
Digital photo frame	4 (2.0%)	
Hoists	5 (2.5%)	
Electronic mattress	5 (2.5%)	
Automatic gas switch-off device	7 (3.0%)	2 (2.4%)
Stove timer	7 (3.0%)	
CCTV	8 (4.0%)	1 (1.2%)
Baby monitors	11 (5.5%)	1 (1.2%)
Riser recliner chair	11 (5.5%)	
Cooker alarm	12 (6.0%)	5 (5.9%)
Bed occupancy sensor	13 (6.5%)	1 (2.4%)
Electronic medicine dispensers	13 (6.5%)	8 (9.4%)
Electronic reminders	13 (6.5%)	2 (2.4%)
Electronic tracking device	14 (7.0%)	12 (14.1%)
Object locator	14 (7.0%)	
Picture button telephone	15 (7.5%)	
Smartwatch	15 (7.5)	1 (1.2%)
Movement detectors/sensors	16 (8.0%)	4 (4.7%)
Audio books	20 (10.0%)	22 (25.9%)
Voice-controlled personal assistant such as Alexa or Siri	21 (10.5%)	3 (3.5%)
Web camera	21 (10.5%)	2 (2.4%)
Smart lights	22 (11.0%)	
Smart plugs	27 (13.5%)	
Door alarm	28 (14.0%)	2 (2.4%)
Electric bed	28 (14.0%)	1 (1.2%)
Falls alarm	31 (15.5%)	6 (7.1%)
Automatic night lamp	32 (16.0%)	1 (1.2%)
GPS tracking device	33 (16.5%)	7 (8.2%)
Large button telephone	37 (18.5%)	8 (9.4%)
Memory clock	42 (21.0%)	7 (8.2%)
Computer/Laptop	52 (26.0%)	10 (11.8%)
Smart gas and electricity meter	56 (28.0%)	2 (2.4%)
Pendant alarm	66 (33.0%)	35 (41.2%)
Electronic day, date and time clock (Dementia clock)	82 (41.0%)	9 (10.6%)
Video communication systems such as Skype or FaceTime	89 (44.5%)	3 (3.5%)
Tablet computer	90 (45.0%)	7 (8.2%)
Smartphone	91 (45.5%)	20 (23.5%)
**Use of Assistive Technology**	N	
Managing day to day spending	8 (4.0%)	
Everyday activities such as eating, washing, dressing, toileting	17 (8.5%)	
Outdoor mobility	24 (12.0%)	
Managing finances	24 (12.0%)	
Indoor mobility	25 (12.5%)	
Reducing effort when you care for someone with dementia	60 (30.0%)	
Leisure	93 (46.5%)	
Memory or reminders	125 (62.5%)	
Communication	132 (66.0%)	
Safety	157 (78.5%)	
**Reason why Assistive Technology is no longer in use:**		
Ethical reasons	3 (3.5%)	
The assistive technology is no longer working	5 (5.9%)	
I or a family member support the person with dementia	11 (12.9%)	
Removed assistive technology as person with dementia no longer wanted it	14 (16.5%)	
Formal/paid carers support the person with dementia	15 (17.6%)	
The assistive technology device has been replaced by a better device	16 (18.8%)	
The person with dementia is no longer able to use it	69 (81.2%)	

### Costs of AT

Carers chose AT predominantly by themselves (43%), followed by carers choosing along with the person with dementia (21%) and then by health or social care professionals choosing the AT (18.9%). Only 7 participants reported that the AT was chosen exclusively by the person with dementia. From participants who reported (*n* = 168), initial costs for the AT ranged from £16.00 to £10,000 (median = £400) and monthly ongoing costs (*n* = 179) ranged from no additional costs to £150.00 a month (median = £17). The monthly costs usually involved expenditure towards pendant alarm subscriptions and monthly phone subscription and internet costs. The AT was purchased predominantly by the participant (54.4%), by another carer (10%), by the person with dementia (23.9%); or provided by social services/health system without a charge (9.7%). Most of the carers rated the AT they were using were either extremely good (35.3%) or somewhat good (49.3%) for value for money. Carers would definitely (62.7%) or probably (31.3%) recommend the use of AT to other carers, with no carers saying they would not recommend AT. There was no significant difference in the initial and monthly costs of AT to the perception of value for money (Table 3).

**Table 3:** Cost of AT and perceived value for money

Independent-samples Kruskal-Wallis test Null Hypothesis	N	Test statistic	Sig.
The distribution of Approximate initial cost of AT is the same across categories of value for money of assistive technology.	168	7.002	0.07
The distribution of Approximate monthly cost of AT is the same across categories of value for money of assistive technology.	178	3.943	0.26

The significance level is .050

### Perceived impact of AT

Carers were asked to choose up to three currently used AT and asked to respond to questions on how the nominated AT helps reduce their effort in caring for someone with dementia, their perceived impact of AT (objective 3) in reducing stress, managing anxiety, reducing harm/ potential harm to the person with dementia and making their caring role easier (Table 4). Carers indicated that the level of care provided to the person with dementia had not changed (48.3%) since the introduction of AT; with some carers reporting that AT improved a little (32.6%) and improved a lot (11.5%) of the care provided. Carers (*n* = 123) felt that AT was very helpful (24.9%) or helpful (21.1.%) in reducing the need for additional paid care. 34.8% of carers reported being extremely satisfied with the AT, with carers using 5 or more AT devices being significantly more satisfied with AT than carers using 4 or less AT devices (*p* = 0.001). There were no significant differences between level of satisfaction with AT to age, living arrangements or relationship with the person with dementia and there was no significant difference between the number of AT being used to the perceived impact on coping with caring and personal relationship with the person with dementia.

**Table 4:** Perceived impact of AT

	% of responses based on AT currently in use				
	Not at all helpful	A little helpful	Quite helpful	Helpful	Very helpful
AT helps in reducing effort (*n* = 200)	8.8	27.2	16.5	25.0	22.5
AT helps in reducing stress (*n* = 194)	5	21.1	11.0	23.9	**39.0**
AT helps in reducing anxiety (*n* = 185)	7.0	19.4	12.0	23.1	**38.6**
AT helps make caring role easier (*n* = 198)	7.2	26.4	10.8	**30.8**	24.8
AT reduces need for additional paid care (*n* = 123)	32.2	11.9	10.0	21.1	**24.8**
AT helps reduce harm/potential harm (*n* = 198)	32.5	16.4	7.4	15.7	28.0
	Deteriorated a lot	Deteriorated a little	Not changed	Improved a little	Improved a lot
Care provided for a person with dementia changed (*n* = 198)	4.0	3.6	**48.3**	32.6	11.5
	Extremely dissatisfied	Somewhat dissatisfied	Neither satisfied/dissatisfied	Somewhat satisfied	Extremely satisfied
Overall satisfaction with AT	1.0	1.0	7.5	55.2	**34.8**
Less than 5 AT used (N)	1.3 (1)	0 (0)	17.3 (13)	54.7 (41)	26.7 (20)
Five or more AT used (N)	0.8 (1)	1.6 (2)	1.6 (2)	56.0 (70)	40.0 (50)
	Value	df	Asymptotic Significance (2-sided)		
Pearson chi-square	19.200	4	**0.001**		

### General physical and mental health of carers

To highlight the general physical and mental wellbeing of carers of persons with dementia who use AT (objective 4), mean PCS and MCS scores were calculated for the SF-12 (Table 5). The mean PCS (*n* = 201) was 49.19 (95% CI 47.75–50.63) and the mean MCS (*n* = 201) was 45.37 (95% CI 43.93–46.80). PCS scores were significantly (*p* < 0.001) associated with age, with older carers having lower PCS scores. MCS scores were also significantly associated with age (*p* = 0.012) with carers in the 46–65 age group having lower MCS scores than other age groups and women having lower MCS scores compared to men (*p* = 0.002). Carers who lived with the person with dementia had significantly (*p* < 0.001) lower PCS scores than those who lived away. Children had better PCS scores than spouses (*p* < 0.001).

**Table 5:** Physical and Mental Health

			PCS		MCS	
		N	Mean	95% CI	Mean	95% CI
SF-12 Scores		201	49.19	47.75–50.63	45.37	43.93–46.80
Age Groups	< 45	20	54.78	52.53–57.02	49.52	45.37–53.68
	46–65	105	51.62	49.81–53.43	43.76	41.70–45.82
	> 66	76	44.37	41.88–46.86	46.49	44.22–48.75
	p		**0.000**		**0.012**	
Sex	Men	65	49.28	46.67–51.89	49.23	47.35–51.10
	Women	131	49.10	47.32–50.88	43.37	41.46–45.29
	p		0.536		**0.002**	
Living arrangements	Living with the person with dementia	103	46.18	43.93–48.43	44.69	42.69–46.69
	Living away from the person with dementia	98	52.36	50.78–53.94	46.08	43.98–48.17
	p		**< 0.001**		0.244	
Marital status	Single	17	52.70	46.77–58.63	41.65	35.49–47.81
	Married/civil partnership	158	49.30	47.71–50.88	46.11	44.53–47.69
	Divorced	22	45.80	40.89–50.71	44.00	39.24–48.76
	Widowed	3	54.08	47.00–61.17	43.42	21.34–65.50
	p		0.063		0.515	
Relationship with person with dementia	Parent	110	51.74	50.08–53.51	44.38	42.34–46.42
	Sibling	3	39.13	9.67–68.58	52.61	48.80–56.42
	Friend	2	57.48	44.27–70.70	51.75	29.26–74.24
	Spouse	72	44.86	42.17–47.54	46.27	44.00–48.54
	Grandparent	3	57.80	50.22–65.37	49.56	24.03–75.10
	Other	10	50.59	44.74–56.44	44.16	35.45–52.88
	p		**< 0.001**		0.436	
Highest level of education	Secondary school	8	47.01	35.10–58.93	40.53	29.03–52.02
	College	58	50.45	47.80–53.10	43.85	41.10–46.60
	Undergraduate university degree	76	47.64	45.16–50.13	47.03	44.88–49.17
	Postgraduate university degree	51	49.81	47.14–52.47	45.38	42.32–48.45
	Other	8	53.07	46.63–59.51	45.28	36.63–53.94
	p		0.302		0.305	
Satisfaction with AT	Extremely satisfied	70	48.71	46.32–51.10	48.26	46.29–50.22
	Not extremely satisfied	130	49.38	47.54–51.21	43.94	42.04–45.85
	P		0.720		**0.010**	
Number of AT being used	Less than 5 AT	76	49.20	46.95–51.46	46.16	43.88–48.44
	5 or more AT	125	49.19	47.30–51.08	44.88	43.02–46.75
	p		0.757		0.561	
Coping with caring	Coped very well	45	54.52	52.85–56.19	49.83	47.41–52.26
	Coped quite well	79	49.38	47.38–51.39	46.86	44.98–48.74
	Coped ok	68	45.16	42.14–48.17	42.45	39.75–45.14
	Coped poorly	9	51.37	42.80–59.95	32.00	21.65–42.35
	p		**0.000**		**0.000**	
Relationship with the person with dementia	Very good	50	51.90	49.85–53.96	48.47	45.99–50.96
	Quite good	107	47.18	45.15–49.21	46.23	44.38–48.09
	Fair	34	51.68	47.72–55.64	40.59	36.83–44.35
	Quite poor	5	45.45	25.56–65.35	35.00	20.23–49.77
	Very poor	3	60.48	53.31–67.65	36.69	0.48–72.90
	p		**0.003**		**0.001**	

MCS scores were significantly higher (*p* = 0.010) in carers who expressed they were extremely satisfied with the AT but there were no significant differences in PCS and MCS scores to the number of AT used. There was a significant relationship between coping with caring (*p* < 0.001) and perceived relationship (*p* < 0.003) with the person with dementia, with those who coped well and had a good relationship with the person with dementia having better PCS and MCS scores.

## Discussion

This study adds to the literature on carers’ experiences of using AT and examines its impact on a sample of carers across the UK. We found carers and persons with dementia use a wide variety and multiple types of AT devices. Smart phones and tablet computers were predominantly used, this is perhaps because they can be used for more than one purpose, such as communication, safety (tracking, medication reminders) and leisure activities. The use of AT largely for safety (monitoring/tracking, movement sensors, pendant alarms, medication management), followed by communication and reminders is similar to earlier findings [33]. We found that age is not a barrier to the number of AT being used, or a reason for abandoning AT, which is dissimilar to previous findings [3] which found that older participants did not perceive AT as useful in caregiving activities and may be reluctant to adopt new technologies. This contradiction could be because the choice of AT does not depend only on the spousal (older) carer; other family members and health care professionals assist with the choice and purchase of AT. In addition, older carers are now comfortable to use technology that are pervasive (such as smart phones and tablet computers) and are now using them as AT [34, 35].

This study also provides information on the range of expenditure on AT currently being used in dementia care. Carers consider AT as good value for money, due to the perception of AT supporting safety and communication (useful for both the carer and the person with dementia) and leisure of the person with dementia. This could support the person with dementia living for longer at home [36]. Carers decide and are willing to abandon AT especially devices such as pendant alarms when it is no longer possible for the person with dementia to use them. This continues to raise the issue of the suitability of AT that are purchased, trialled, and then abandoned - increasing waste both economically for carers and environmentally. To avoid this, focus of care for a person with dementia should be seen as a long-term condition, with an unpredictable progression. Health care providers, carers and persons with dementia would benefit from a centrally-funded access point to high-quality information on AT (this is currently only being provided by charities/third sector organisations) and establishing local loan stores for AT [21]. Interestingly ethical reasons are not highlighted as a prime reason for abandoning AT, this could be because carers feel the overriding reasons of safety and welfare of the person with dementia is more important [16, 37].

Caring could be a positive experience for some but it does have an influence on anxiety, stress and fatigue, as well as causing problems with physical health among family carers [38]. None of the carers in our study, felt they could not recommend the AT to other carers; this could be because carers, irrespective of perceived impact and personal satisfaction with the AT, felt AT enhances and/or supplements care for a person with dementia and their own quality of life. In this survey, carers report that AT had an impact on caring. We found, carers felt no change in the effort of caring since they started using AT, this could be because the progress of dementia is often slow and unpredictable, and perhaps AT is helping to maintain the status quo of the needs of a person with dementia; in addition, the care could also be supplemented by support from formal carers. Carers felt that AT helps in reducing their stress and anxiety, this could be because AT devices for communication and safety provides a sense of reassurance for carers, especially when they live away from the person with dementia. Carers did not perceive AT as being useful in reducing harm, this may be because AT such as smart phones and tablet computers are seen as devices that facilitate communication and leisure, rather than safety and welfare. Carers also felt that AT helped reduce the need for additional paid care, whilst our study did not quantify this, it should be an area for further investigation into the economic advantages of using AT. This study also reveals that, larger the number of AT being used the greater the satisfaction with AT. While this needs to be further explored, one reason could be because carers may be sequentially adding AT, i.e. using additional AT one at a time, based on the changing needs of the person with dementia and hence could be more satisfied when the additional AT meets their needs.

Previous research [9, 33, 39] has shown that there were fewer number of AT that met the needs of basic activities of daily living such as eating and bathing etc., which is confirmed by our survey. Basic activities of daily living, could be the tasks that a person with dementia is most dependent on [39] and carers living with persons with dementia are likely to be spousal carers and living with the person with dementia would mean carers being called on to physically assist them more often, this could explain why PCS scores were lower for carers living with the person with dementia. It has been reported earlier [40, 41] that coping with care responsibilities and the nature of the relationship with the person with dementia has a direct effect on carer quality of life and this is confirmed in our survey.

Carer-person with dementia relationship, carer physical and emotional well-being and coping with the demands of caring are all dimensions of quality of life of family carers [42–44]. This survey provides an insight into the quality of life of carers who are currently using AT. Older carers had lower PCS scores similar to other studies [3, 45], this could also explain why spousal carers had lower PCS scores, than children who care for persons with dementia. MCS scores for carers in the 46–65 age group was significantly lower, this could be because carers within this age group are likely to be the “sandwich” generation [38], with responsibilities for caring for their own children and families whilst having to care for their aged parents.

Carers who felt extremely satisfied with AT had better MCS scores, and this could be because, using the AT for reassurance (safety, communication) might be more important for carer quality of life. The amount of informal care provided to older people with disabilities in England is unlikely to keep pace with demand [6], this disconnect has been sharply highlighted in restrictions and increased reliance on technology brought about by the covid-19 lockdown [14, 46, 47]. For dementia care, as this study shows, AT may help, by making the caring role easier and reducing the effort required for caring for a person with dementia. This could be why carers who felt that their relationship and ability to cope with caring for a person with dementia had better PCS and MCS scores.

### Implications for practice, policy and research

Increasing amount of care for persons with dementia is being provided by carers. AT manufacturers and healthcare professionals advocate AT as a solution to decrease the amount of formal care provided for persons with dementia, which raises important issues in long-term dementia care. Assessment and use of AT by carers of persons with dementia, should consider dementia as a long-term condition and move away from applying the existing ‘acute care’ model [21, 36, 48] of brief interventions and rigid protocols (such as providing pendant alarms to all persons at risk of falls) to a fundamentally unpredictable condition [49]. AT providers and healthcare professionals could use a survey tool such as the CATEQ periodically to sense check the types, uses and impact of AT and modify development of new AT and provision of AT to people with dementia. Policymakers should undertake a concerted effort to provide a centrally funded access point to high-quality information on AT and consider set-up and expanding use of a loan store for AT devices for dementia. Policies should be formulated to support research and Government backing to provide focused funding that can capitalise on emerging artificial intelligence driven technology that are incorporated into smart phones and tablet computers, that could provide further support for carers and persons with dementia. Further research is needed to find what if any influence the incremental addition of AT can have on satisfaction and impact on carer quality of life and the need for additional formal/paid care. Better integration of various types of AT into fewer AT devices and cost-benefit analysis of AT use in reducing the need for formal/paid care or longer community living should also be explored.

### Strength and limitations

This study provides insight into the current use and impact of AT in dementia care across the UK. This questionnaire covered items related to uses, costs, impact and satisfaction giving a much better understanding of carers’ experience with AT. One of the limitations for this study is that the questionnaire was in English and despite intensive efforts, we could not increase participation from carers from a non-white ethnic background. This has meant our sample may not fully represent the multicultural UK population. This survey was launched just before the lockdown and subsequent restrictions due to Covid-19 in the UK, which could have resulted in inability of some carers to respond to the survey, for e.g. not able to go out due to shielding, to post paper questionnaires or increased caring responsibilities due to reduced services or other support. This could have led to fewer responses from carers who are using AT to look after the most vulnerable of persons with dementia.

## Conclusions

This study provides information on carers’ experiences with using AT in dementia care. Safety, memory, and communication are areas where AT are most used. Our study finds that AT can support carers in their efforts in caring for someone with dementia. Carers report that AT helps them in reducing effort, making the caring role easier and reducing their stress and anxiety. However, additional support is needed for carers in the choice, purchase and continued use of AT. Incremental use of AT and cost-effectiveness of AT in supporting the person with dementia and their carers are areas where future service development and research should target. Viewing dementia as a long-term condition with planned use of AT as a solution in the care pathway could improve the experience of dementia care at home.

## Supplementary Information

**Additional file 1.**

## Data Availability

The datasets generated during the study are available from the corresponding author on reasonable request.

## Abbreviations

AT: Assistive technology
CATEQ: Carers Assistive Technology Experience Questionnaire
CI: Confidence interval
MCS: Mental component score of the short form health survey
OxDARE: Oxford dementia and aging research database
PCS: Physical component score of the short form health survey
SF-12: Short form health survey 12
SD: Standard deviation
