# Supplementary material for: Carers’ experiences of assistive technology use in dementia care: a cross sectional survey
Source: BMC Geriatr. 2021 Aug 25;21:471. doi: 10.1186/s12877-021-02417-1 (PMC8385483; doi:10.1186/s12877-021-02417-1)
Supplement: Supplementary file 1 — Additional file 1. [file 12877_2021_2417_MOESM1_ESM.pdf]

Carers’ Assistive Technology Experience Questionnaire

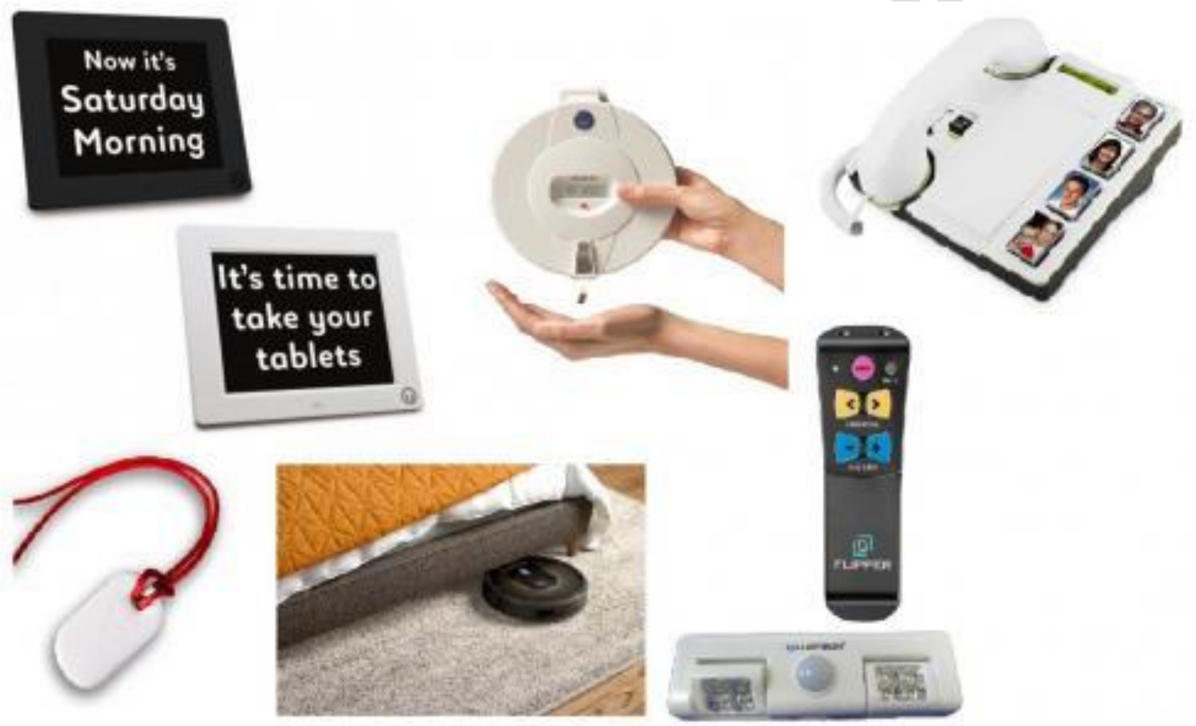

Please send back the completed questionnaire in the reply-paid envelope.

Return Address: Vimal Sriram, DPhil student, Health Services Research Unit, Nuffield Department of Population Health, Richard Doll Building, Old Road Campus, University of Oxford, Oxford – OX3 7LF

DO NOT COPY

Carers’ Assistive Technology Experience Questionnaire

General Information:
You are being invited to take part in the research study ‘Carers' experience of Assistive Technology (AT) use in dementia’.
Assistive Technology (AT) are products such as talking clocks, electronic medication dispensers, robotic vacuum cleaners, smart gas meters, communication books, GPS navigation systems, falls and motion detectors, smartphone apps and door exit alarms.

You can take part in this survey if you are:

- Aged 18 years or above
- A family member/friend/neighbour looking after a person with dementia
- Providing at least 10 hours care (such as supporting shopping, leisure, personal care, finance) to the person with dementia per week at home
- Using/used at least one AT device at home within the past year

It should take you about 30 minutes to finish this survey. The purpose of this research is to find out: the benefits and disadvantages of AT; the facilitators or barriers to using AT; and the impact of using AT on carer wellbeing.

Do I have to take part?
Please note that your participation is voluntary. If you do decide to take part, you may withdraw at any point during the survey for any reason and you can simply choose not to send the questionnaire back to us.

How will my data be used?
Your answers will be completely anonymous, unless you provide your email address at the end of the survey to be contacted for an interview. We will take all reasonable measures to ensure that data remain confidential.
Your answers will be entered, stored and examined using University of Oxford approved software. All data will be stored in a password-protected file and may be used in academic publications. All the questions in this survey are optional. Research data will be stored for a minimum of three years after publication or public release.

Your email address (if provided) will only be used to contact you with a view to taking part in an interview at a later date. It will be deleted once the interview has concluded or if you tell us you no longer wish to be interviewed.

The data that we collect from you may be transferred to, stored and/or processed at a destination outside the European Economic Area ("EEA"), including in the UK. By submitting your personal data, you agree to this transfer, storing or processing.

Who will have access to my data?
The University of Oxford is the data controller with respect to your personal data, and as such will determine how your personal data is used in the study. The University will process your personal data for the purpose of the research outlined above. Research is a task that we perform in the public interest. Further information about your rights with

DO NOT COPY

respect to your personal data is available at https://compliance.admin.ox.ac.uk/individual-rights

We would also like your permission to use your anonymised data in future studies, and to share data with other researchers (e.g. in online databases). Any personal information that could identify you will be removed or changed before files are shared with other researchers or results are made public.

Responsible members of the University of Oxford and funders may be given access to data for monitoring and/or audit of the study to ensure we are complying with guidelines, or as otherwise required by law.

This survey is for a DPhil (doctoral) research project. The Principal Researcher is Vimal Sriram, who is attached to the Nuffield Department of Population Health at the University of Oxford. This project is being completed under the supervision of Professor Crispin Jenkinson and Associate Professor Michele Peters.

This project has been reviewed by, and received ethics clearance through, the University of Oxford Central University Research Ethics Committee [R57703/RE002].

Who do I contact if I have a concern about the study or I wish to complain?
If you have a concern about any aspect of this study, please contact Vimal Sriram, Nuffield Department of Population Health, Richard Doll Building, Old Road Campus Oxford OX3 7LF, UK on 01865 743762 or vimal.sriram@dph.ox.ac.uk or the supervisors Dr Michele Peters (michele.peters@ndph.ox.ac.uk) or Professor Crispin Jenkinson (crispin.jenkinson@ndph.ox.ac.uk) and we will do our best to answer your query. I will acknowledge your concern within 10 working days and give you an indication of how it will be dealt with. If you remain unhappy or wish to make a formal complaint, please contact the Chair of the Medical Sciences Interdivisional Research Ethics Committee at the University of Oxford who will seek to resolve the matter as soon as possible:
Email: ethics@medsci.ox.ac.uk; Address: Research Services, University of Oxford, Wellington Square, Oxford OX1 2JD. The Chair will seek to resolve the matter in a reasonably expeditious manner.

CONSENT:
Please select your choice below and place a tick (√) in the appropriate box.

Ticking on the "agree" button below indicates that:
- You have read the above information
- You voluntarily agree to participate
- You are at least 18 years of age

If you do not wish to participate in the research study, please decline participation by ticking on the "disagree" box or you can simply choose not to send the questionnaire back to us.

○ Agree

○ Disagree

DO NOT COPY

There are three sections to this questionnaire.

The first section will ask you about the Assistive Technology (AT) that you are currently using and what you are using it for. We will ask about AT devices that you may have used before and are no longer using, later in the questionnaire.

The second section will ask you questions about your experiences of using the AT and its impact on your daily life.

The last section will ask you questions about yourself.

Section 1: Assistive Technology

Q1 What assistive technology are you or the person with dementia who you support and care for currently using? (please select all that apply)

- ☐ Assistive Robots
- ☐ Audio books
- ☐ Automatic gas switch-off device
- ☐ Automatic night lamp
- ☐ Baby monitors
- ☐ Bed occupancy sensor
- ☐ CCTV
- ☐ Computer/Laptop
- ☐ Cooker alarm
- ☐ Door alarm
- ☐ Electric bed
- ☐ Electronic day, date and time clock (Dementia clock)
- ☐ Electronic medicine dispensers
- ☐ Electronic reminders
- ☐ Electronic tracking device
- ☐ Falls alarm
- ☐ Flood detector
- ☐ GPS tracking device
- ☐ Large button telephone
- ☐ Memory clock
- ☐ Movement detectors/sensors
- ☐ Object locator
- ☐ Pendant alarm
- ☐ Picture button telephone
- ☐ Robotic pet(s)
- ☐ Smart gas and electricity meter
- ☐ Smart lights
- ☐ Smartphone
- ☐ Smart plugs
- ☐ Smartwatch
- ☐ Stove timer
- ☐ Tablet computer
- ☐ Video communication systems such as Skype or FaceTime
- ☐ Voice-controlled personal assistant such as Alexa or Siri
- ☐ Web camera

DO NOT COPY

Q2 If you use any other assistive technology not listed, can you provide a list here?

____

____

Q3 What do you use the assistive technology for? (please select all that apply)

- ☐ Everyday activities such as eating, washing, dressing, toileting
- ☐ Indoor mobility
- ☐ Outdoor mobility
- ☐ Communication
- ☐ Memory or reminders
- ☐ Leisure
- ☐ Safety
- ☐ Managing day to day spending
- ☐ Managing finances
- ☐ Reducing effort when you care for someone with dementia
- ☐ Other ____

DO NOT COPY

Q4 Who was involved in choosing the assistive technology <u>currently</u> used? (please select all that apply)

- ☐ The person with dementia
- ☐ Myself
- ☐ Myself together with the person with dementia
- ☐ Another carer (family, friend, neighbor)
- ☐ Health or social care professionals (Doctor, Nurse, Occupational Therapist, Social worker etc.)
- ☐ Other ____

Q5 Who paid for the assistive technology <u>currently</u> used? (please select all that apply)

- ☐ The person with dementia
- ☐ Myself
- ☐ Another carer (family, friend, neighbor)
- ☐ The assistive technology was provided by social services without a charge
- ☐ The assistive technology was provided by a charity
- ☐ Other ____

Q6 Can you give the approximate cost (in pounds) of initial buying of the assistive technology <u>currently used,</u> paid for by the person with dementia or by you or another carer (family, friend or neighbour)?

____

____

____

Q7 Can you give the approximate cost (in pounds) of monthly ongoing costs of the assistive technology <u>currently used,</u> paid for by the person with dementia or by you or another carer (family, friend or neighbour)?

____

____

____

DO NOT COPY

Section 2: Using Assistive Technology

Q8 Please list **up to three** assistive technology devices that you are <u>currently</u> using. If you currently use only one or two devices list those.

Assistive Technology 1 ____

Assistive Technology 2 ____

Assistive Technology 3 ____

Answer the next set of questions on how you felt in your caring role in the past month using your selected assistive technology devices.

There might have been good and bad days, but we want to capture a snapshot of how you have felt on a typical day during the past month.

For each question below, choose the most appropriate option for the assistive technology device(s) you have chosen above, that best describes how you felt and select that option.

Q9 How involved are you in the on-going use of assistive technology?

	The person with dementia uses the assistive technology independently	I assist the person with dementia to use the assistive technology	The person with dementia and I use the assistive technology together	I use the assistive technology by myself
Assistive Technology 1	○	○	○	○
Assistive Technology 2	○	○	○	○
Assistive Technology 3	○	○	○	○

DO NOT COPY

Q10 How often do you have to help the person with dementia to use the assistive technology?

	Never	Rarely	Sometimes	Often	Always	Need never arose
Assistive Technology 1	○	○	○	○	○	○
Assistive Technology 2	○	○	○	○	○	○
Assistive Technology 3	○	○	○	○	○	○

Q11 Are you concerned about the cost of the assistive technology?

	Never	Rarely	Sometimes	Often	Always	Not applicable
Assistive Technology 1	○	○	○	○	○	○
Assistive Technology 2	○	○	○	○	○	○
Assistive Technology 3	○	○	○	○	○	○

Q12 How helpful is the assistive technology in maintaining dignity of the person with dementia?

	Not at all helpful	A little helpful	Quite helpful	Helpful	Very helpful	Does not apply
Assistive Technology 1	○	○	○	○	○	○
Assistive Technology 2	○	○	○	○	○	○
Assistive Technology 3	○	○	○	○	○	○

DO NOT COPY

Q13 How concerned are you about your privacy and confidentiality when using assistive technology?

	A great deal	A lot	A moderate amount	A little	Not at all	Does not apply
Assistive Technology 1	○	○	○	○	○	○
Assistive Technology 2	○	○	○	○	○	○
Assistive Technology 3	○	○	○	○	○	○

Q14 How concerned are you about privacy and confidentiality of the person with dementia when using assistive technology?

	A great deal	A lot	A moderate amount	A little	Not at all	Does not apply
Assistive Technology 1	○	○	○	○	○	○
Assistive Technology 2	○	○	○	○	○	○
Assistive Technology 3	○	○	○	○	○	○

Q15 How helpful is the assistive technology in better managing the space/living arrangements for the person with dementia?

	Not at all helpful	A little helpful	Quite helpful	Helpful	Very helpful	Does not apply
Assistive Technology 1	○	○	○	○	○	○
Assistive Technology 2	○	○	○	○	○	○
Assistive Technology 3	○	○	○	○	○	○

DO NOT COPY

Q16 To what extent does the assistive technology meet your needs?

	A great deal	A lot	A moderate amount	A little	Not at all
Assistive Technology 1	○	○	○	○	○
Assistive Technology 2	○	○	○	○	○
Assistive Technology 3	○	○	○	○	○

DO NOT COPY

Section 2: Impact of using Assistive Technology

Q17 What input is required from you to use the assistive technology (please select all that apply)?

	Physical assistance	Verbal encouragement	Reminders and teaching	Monitoring	Responding to alerts	Maintaining assistive technology when it is not working	Removing assistive technology when it is not working or dangerous
Assistive Technology 1	○	○	○	○	○	○	○
Assistive Technology 2	○	○	○	○	○	○	○
Assistive Technology 3	○	○	○	○	○	○	○

Q18 How often are you able to solve problems with the assistive technology by yourself?

	Never	Rarely	Sometimes	Often	Always	Need never arose
Assistive Technology 1	○	○	○	○	○	○
Assistive Technology 2	○	○	○	○	○	○
Assistive Technology 3	○	○	○	○	○	○

Q19 How helpful is the assistive technology in reducing your effort in caring for someone with dementia?

	Not at all helpful	A little helpful	Quite helpful	Helpful	Very helpful
Assistive Technology 1	○	○	○	○	○
Assistive Technology 2	○	○	○	○	○
Assistive Technology 3	○	○	○	○	○

Q20 How helpful is the assistive technology in reducing your stress?

	Not at all helpful	A little helpful	Quite helpful	Helpful	Very helpful	Does not apply
Assistive Technology 1	○	○	○	○	○	○
Assistive Technology 2	○	○	○	○	○	○
Assistive Technology 3	○	○	○	○	○	○

DO NOT COPY

Q21 How helpful is the assistive technology in managing your anxiety?

	Not at all helpful	A little helpful	Quite helpful	Helpful	Very helpful	Does not apply
Assistive Technology 1	○	○	○	○	○	○
Assistive Technology 2	○	○	○	○	○	○
Assistive Technology 3	○	○	○	○	○	○

Q22 How helpful is the assistive technology in making your caring role easier?

	Not at all helpful	A little helpful	Quite helpful	Helpful	Very helpful
Assistive Technology 1	○	○	○	○	○
Assistive Technology 2	○	○	○	○	○
Assistive Technology 3	○	○	○	○	○

Q23 How helpful is the assistive technology in giving you additional time for tasks that you have to do?

	Not at all helpful	A little helpful	Quite helpful	Helpful	Very helpful	Does not apply
Assistive Technology 1	○	○	○	○	○	○
Assistive Technology 2	○	○	○	○	○	○
Assistive Technology 3	○	○	○	○	○	○

DO NOT COPY

Q24 How helpful is the assistive technology in giving you more time for yourself?

	Not at all helpful	A little helpful	Quite helpful	Helpful	Very helpful
Assistive Technology 1	○	○	○	○	○
Assistive Technology 2	○	○	○	○	○
Assistive Technology 3	○	○	○	○	○

Q25 How helpful is the assistive technology in reducing harm/potential harm to the person with dementia?

	Not at all helpful	A little helpful	Quite helpful	Helpful	Very helpful
Assistive Technology 1	○	○	○	○	○
Assistive Technology 2	○	○	○	○	○
Assistive Technology 3	○	○	○	○	○

Q26 How helpful is the assistive technology in reducing the need for additional paid care?

	Not at all helpful	A little helpful	Quite helpful	Helpful	Very helpful	Does not apply
Assistive Technology 1	○	○	○	○	○	○
Assistive Technology 2	○	○	○	○	○	○
Assistive Technology 3	○	○	○	○	○	○

DO NOT COPY

Q27 Since you started using the assistive technology, how has the care you provide for the person with dementia changed?

	Improved a lot	Improved a little	Not changed	Deteriorated a little	Deteriorated a lot
Assistive Technology 1	○	○	○	○	○
Assistive Technology 2	○	○	○	○	○
Assistive Technology 3	○	○	○	○	○

Q28 How would you rate the value for money of assistive technology?

☐ Extremely good

☐ Somewhat good

☐ Neither good nor bad

☐ Somewhat bad

☐ Extremely bad

Q29 Would you recommend assistive technology to other carers?

☐ Definitely

☐ Probably

☐ Unsure

☐ Probably not

☐ Definitely not

Q30 Overall how would you say your satisfaction with assistive technology has been?

☐ Extremely satisfied

☐ Somewhat satisfied

☐ Neither satisfied nor dissatisfied

☐ Somewhat dissatisfied

☐ Extremely dissatisfied

DO NOT COPY

Section 2: Previously used Assistive Technology

Q31 Are there assistive technology devices that you have previously used and are no longer using?

☐ Yes

☐ No

Q32 If Yes, which assistive technology are you no longer using? (please select all that apply)

☐ Assistive Robots

☐ Audio books

☐ Automatic gas switch-off device

☐ Automatic night lamp

☐ Baby monitors

☐ Bed occupancy sensor

☐ CCTV

☐ Computer/Laptop

☐ Cooker alarm

☐ Door alarm

☐ Electric bed

☐ Electronic day, date and time clock (Dementia clock)

☐ Electronic medicine dispensers

☐ Electronic reminders

☐ Electronic tracking device

☐ Falls alarm

☐ Flood detector

☐ GPS tracking device

☐ Large button telephone

☐ Memory clock

☐ Movement detectors/sensors

☐ Object locator

☐ Pendant alarm

☐ Picture button telephone

☐ Robotic pet(s)

☐ Smart gas and electricity meter

☐ Smart lights

☐ Smartphone

☐ Smart plugs

☐ Smartwatch

☐ Stove timer

☐ Tablet computer

☐ Video communication systems such as Skype or Face time

☐ Voice-controlled personal assistant such as Alexa or Siri

☐ Web camera

☐ Other____

DO NOT COPY

Q33 When you were using the assistive technology, what did you use it for? (please select all that apply)

- ☐ Everyday activities such as eating, washing, dressing, toileting
- ☐ Indoor mobility
- ☐ Outdoor mobility
- ☐ Communication
- ☐ Memory or reminders
- ☐ Leisure
- ☐ Safety
- ☐ Managing day to day spending
- ☐ Managing finances
- ☐ Reducing effort when you care for someone with dementia

☐ Other ____

Q34 Why are you no longer using the assistive technology? (please select all that apply)

- ☐ The person with dementia is no longer able to use it
- ☐ The assistive technology is no longer working
- ☐ The assistive technology device has been replaced by a better device
- ☐ I, or a family member support the person with dementia
- ☐ Formal/paid carers support the person with dementia
- ☐ Removed assistive technology as it was dangerous
- ☐ Removed assistive technology as person with dementia no longer wanted it
- ☐ Ethical reasons
- ☐ Other

Q35 If you chose other, can you give some detail why you are no longer using the assistive technology?

____

____

____

DO NOT COPY

Section 3: About You

The following questions asks for some basic information about you and your health. This information will help us to see how the experience of using assistive technology varies between different people. All information will be treated in the strictest confidence.

Q36 In general would you say your health is:

- ☐ Excellent
- ☐ Very good
- ☐ Good
- ☐ Fair
- ☐ Poor

Q37 On a typical day, does your health now limit you in moderate activities such as moving a table, pushing a vacuum cleaner, bowling or playing golf? If so, how much?

- ☐ Yes, limited a lot
- ☐ Yes, limited a little
- ☐ No, not limited at all

Q38 On a typical day, does your health now limit you in climbing several flights of stairs? If so, how much?

- ☐ Yes, limited a lot
- ☐ Yes, limited a little
- ☐ No, not limited at all

Q39 During the past four weeks, have you had any problems with your work or other regular daily activities as a result of your physical health and accomplished less than you would like?

- ☐ Yes
- ☐ No

DO NOT COPY

Q40 During the past four weeks, have you had any problems with your work or other regular daily activities as a result of your physical health and were limited in the kind of work or other activities?

☐ Yes

☐ No

Q41 During the past four weeks, have you had any problems with your work or other daily activities as a result of any emotional problems (such as feeling depressed or anxious) and accomplished less than you would like?

☐ Yes

☐ No

Q42 During the past four weeks, have you had any problems with your work or other daily activities as a result of any emotional problems (such as feeling depressed or anxious) and did work or other activities less carefully than usual?

☐ Yes

☐ No

Q43 During the past four weeks, how much did pain interfere with your normal work (including work both outside the home and housework)?

☐ Not at all

☐ A little bit

☐ Moderately

☐ Quite a bit

☐ Extremely

DO NOT COPY

Q44 How much of the time during the past four weeks have you felt calm and peaceful?

- ☐ All of the time
- ☐ Most of the time
- ☐ Some of the time
- ☐ A little of the time
- ☐ None of the time

Q45 How much of the time during the past four weeks did you have a lot of energy?

- ☐ All of the time
- ☐ Most of the time
- ☐ Some of the time
- ☐ A little of the time
- ☐ None of the time

Q46 How much of the time during the past four weeks have you felt downhearted and blue?

- ☐ All of the time
- ☐ Most of the time
- ☐ Some of the time
- ☐ A little of the time
- ☐ None of the time

Q47 During the last four weeks, how much of the time has your physical health or emotional problems interfered with your social activities (like visiting with friends, relatives, etc.)?

- ☐ All of the time
- ☐ Most of the time
- ☐ Some of the time
- ☐ A little of the time
- ☐ None of the time

DO NOT COPY

Q48 During the past four weeks, thinking of the physical and emotional demands of caring, do you feel that they have had…

- ☐ No impact on your health
- ☐ Little impact on your health
- ☐ Some impact on your health
- ☐ A significant impact on your health
- ☐ A very significant impact on your health

Q49 During the past four weeks, considering all the demands that caring places on you, do you feel overall that you have…

- ☐ Coped very well
- ☐ Coped quite well
- ☐ Coped OK
- ☐ Coped quite poorly
- ☐ Coped very poorly

Q50 Your relationship with the person with dementia over the last four weeks has been…

- ☐ Very good
- ☐ Quite good
- ☐ Fair
- ☐ Quite poor
- ☐ Very poor

Q51 How would you describe your gender?

- ☐ Male
- ☐ Female
- ☐ Prefer to self-describe as________________________________

Q52 What is your age in years?

____

DO NOT COPY

Q53 Which of these best describes what you are doing at present? (please tick all that apply)

☐ Full-time paid work (30 hours or more each week)

☐ Part-time paid work (under 30 hours each week)

☐ Full-time education at school, college or university

☐ Part-time education at school, college or university

☐ Self-employed

☐ Permanently sick or disabled

☐ Fully retired from work

☐ Looking after the home

☐ Voluntary/Charity work

☐ Unemployed

☐ Doing something else (please specify)

____

Q54 What is your legal marital status or same-sex civil partnership status?

☐ Single (never married and never formed a civil partnership)

☐ Married / living as married / in a civil partnership

☐ Separated (but still legally married / in civil partnership)

☐ Divorced / legally dissolved civil partnership

☐ Widowed / surviving partner from civil partnership

Q55 What is your ethnic group?

☐ White

☐ Mixed/Multiple ethnic groups

☐ Indian/Indian British

☐ Pakistani/Bangladeshi/ Pakistani or Bangladeshi British

☐ Black/African/Black or African British

☐ Caribbean/Caribbean British

☐ Chinese/Chinese British

☐ Other Ethnic group (please specify)

____

DO NOT COPY

Q56 What is the highest level of education you have completed?

- ☐ Secondary school
- ☐ College (further education)
- ☐ Undergraduate university degree
- ☐ Postgraduate university degree
- ☐ Other (please specify)

____

Q57 To help assess the financial impact of using assistive technology, it is necessary to ask you for details of your family income, if you wish to disclose it.

- ☐ Less than £10,000
- ☐ £10,000 - £20,000
- ☐ £20,001 - £30,000
- ☐ £30,001 - £ 40,000
- ☐ £40,001 - £50,000
- ☐ £50,001 - £60,000
- ☐ £60,001 - £70,000
- ☐ More than £70,000
- ☐ I do not wish to say

Q58 What are your living arrangements with the person with dementia you provide care for?

- ☐ I live with the person with dementia
- ☐ The person with dementia lives with me
- ☐ I live away from the person with dementia but visit at least daily
- ☐ I live away from the person with dementia but visit at least weekly
- ☐ I live away from the person with dementia and visit at least monthly
- ☐ I have other arrangements (please specify)

____

DO NOT COPY

Q59 The person with dementia is my

- ☐ Parent
- ☐ Child
- ☐ Sibling
- ☐ Friend
- ☐ Neighbour
- ☐ Other (please specify) ________________________________

Q60 We would like to interview some participants from this survey. If you are happy to be invited for an interview, please indicate that below and provide us with your contact details.

- ◯ Yes, I am happy to be contacted about an interview
- ◯ No, I do not want to be contacted about an interview

DO NOT COPY

Q61 If Yes, please provide your email address and/or telephone number below. This information will only be used to contact you about your participation in an interview and will not be shared with anyone outside the research team.

____

Thank you for your time in completing the survey. After analysis, a summary of the results from this survey will be available on the study website: http://bit.ly/atindementia

DO NOT COPY
